# Primary Angiosarcoma of Urinary Bladder: 13th Reported Patient

**DOI:** 10.1155/2015/652870

**Published:** 2015-01-26

**Authors:** Zaher Bahouth, Ismael Masarwa, Sarel Halachmi, Ofer Nativ

**Affiliations:** ^1^Bnai-Zion Medical Center, 3339313 Haifa, Israel; ^2^Faculty of Medicine, Technion Institute of Technology, 3200003 Haifa, Israel

## Abstract

Angiosarcoma of the urinary bladder is an extremely rare and poorly characterized tumor. We are presenting the 13th reported patient who was an 89-year-old man initially presented with massive hematuria. His past medical history included external-beam radiation for prostate cancer 12 years ago. His PSA was 0.26 ng/dL. His CT-Urography demonstrated a highly vascular mass originating from the bladder base. The mass was partially resected, transurethrally. The pathology was consistent with primary angiosarcoma of the urinary bladder. Bone scan and CT-U showed metastasis to spine. The patient was treated with palliative radiotherapy for back pain due to metastasis, and he refused chemotherapy. The patient died 3 months after his initial diagnosis.

## 1. Introduction

Nonurothelial tumors of the urinary bladder represent less than 5% of all bladder tumors, with sarcoma being the most common of which [1]. Angiosarcoma, one of the rarest types of sarcoma, arises from the endothelial layer of blood vessels. It is a highly aggressive cancer and carries very poor prognosis. Only 12 cases of primary angiosarcoma of the urinary bladder have been reported so far [2].

## 2. Case Presentation

### 2.1. The Patient

**Figure 1:**
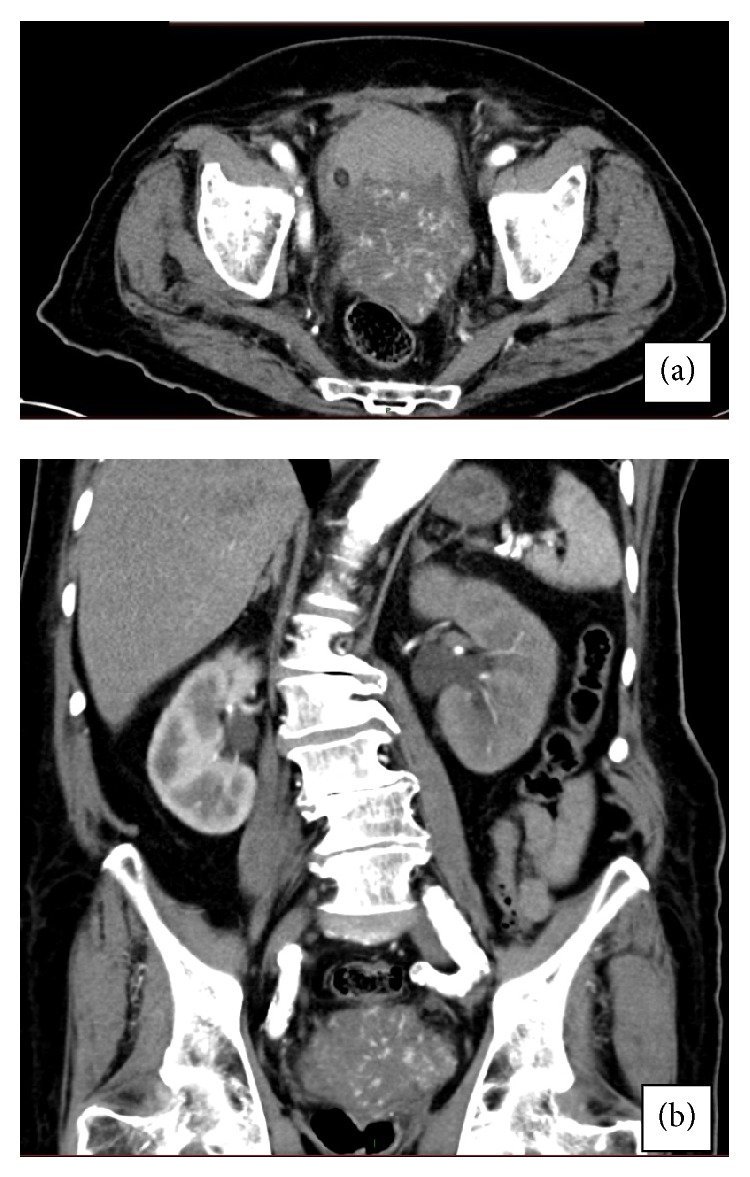
Computed tomography of our patient. (a) Highly vascular mass originating from the left bladder wall, but a prostatic origin could not be absolutely rolled out by CT. (b) Hydronephrosis on the left side and coronal view of the mass.

An 89-year-old male presented to ER with massive hematuria. Two large caliber intravenous cannulas were placed and blood tests for CBC, biochemistry, and blood type were taken. A 3-way 22Fr catheter was urethrally inserted and bladder irrigation with normal saline started. His past medical history included T1 prostatic adenocarcinoma for which he received external beam radiotherapy 12 years ago. His physical examination and digital rectal examination were unremarkable. His creatinine was 0.9 mg/dL, PSA was 0.26 ng/mL, and cytology was negative for malignant cells. CT-Urography demonstrated a large highly vascular mass with irregular margins originating from the left bladder wall (Figure 1(a)). Computed tomography also showed severe left-sided hydronephrosis and hydroureter (Figure 1(b)) and lytic lesions in the vertebrae and pelvic bones suspected as metastases. Cystoscopy showed a large solid mass arising from the bladder base which was resected transurethrally.

### 2.2. Histopathology

**Figure 2:**
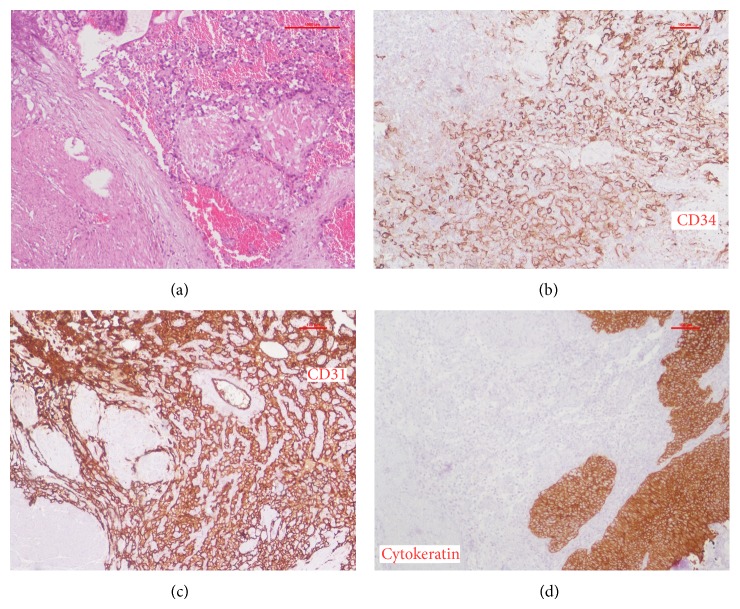
Histopathology and immunohistochemistry. (a) Typical appearance of anastomosing vascular channels as seen with H&E. (b) Tumor cells stain positive for CD34. (c) Tumor cells stain positive for CD31. (d) Tumor cells do not stain for cytokeratin.

Macroscopically, the specimen was 6 × 5 × 4 cm^3^ of grey tissue and blood clots. Microscopically, the tissue diagnosis was consistent with primary angiosarcoma of urinary bladder invading muscularis layer, with anastomosing vascular channels (Figure 2(a)). The tumor strongly stained for CD31 and CD34 and focally for fVIII (Figures 2(b) and 2(c)), three markers for endothelial cells, and was negative for Keratin P63, a marker of epithelial cells (Figure 2(d)). No prostatic tissue was found in the specimen.

### 2.3. Treatment

The patient was referred to palliative radiotherapy because of symptomatic spinal metastasis. The patient was consulted about the option of chemotherapy but he denied. The patient died 3 months after the initial diagnosis.

## 3. Discussion

Nonurothelial neoplasms of the urinary bladder are extremely rare. Of these neoplasms, sarcoma represents the most common nonurothelial neoplasm of the urinary bladder [1]. The most common sarcoma of the bladder is leiomyosarcoma, which accounts for more than half of the sarcomas [1]. Angiosarcoma of the urinary bladder is a very rare subtype of sarcoma with only 12 reported cases [2]. All 12 cases reported presented with hematuria, 11 with gross hematuria, and one with microscopic hematuria. Almost all cases were muscle-invasive tumors. Twelve patients' characteristics are summarized in Table 1. Histological and immunohistochemical features are summarized in Table 2.

Four cases of secondary angiosarcoma of the bladder were previously reported, originating from the penis [3], pelvis [4], Kaposi's sarcoma [5], and vagina [6].

Angiosarcoma is a very aggressive tumor carrying poor prognosis [7]. Most of the angiosarcomas are at least muscle-invasive when diagnosed [8]. Hematuria, the most common presenting symptom, may be life-threatening. Histolopathology mostly establishes the diagnosis, with typical microscopic features and immunohistochemical staining [8]. Cytology may be helpful in some cases [9].

Prior radiation is a well-documented risk factor [4] and should raise a suspicion of angiosarcoma in patients with hematuria and no evidence of urothelial carcinoma. Of the 13 angiosarcoma cases reported, including the one we are reporting, 38% were related to prior exposure to radiotherapy.

Because of the small number of patients reported, there is no accepted gold-standard treatment. Suggested treatment options include chemotherapy, radiotherapy, and radical surgery, with some sort of combinations [2, 8].

## Figures and Tables

**Table 1:** All reported patients' characteristics.

Patient	Age/sex	Etiology	Presentation	Author/year
1	54/M	Hemangioma	Hematuria and obstruction	Jungano, 1907 [10]
2	85/F	Hemangioma	Hematuria, dysuria, and weight loss	Casal et al., 1970 [11]
3	68/M	Primary	Hematuria	Stroup and Chang 1987 [12]
4	78/M	Primary	Hematuria, dysuria	Aragona et al., 1991 [13]
5	55/M	Primary	Hematuria	Ravi, 1993 [14]
6	78/M	RT 13 years prior to Dx	Hematuria	Navon et al., 1997 [15]
7	47/M	Primary	Hematuria, flank pain, and suprapubic pain	Engel et al., 1998 [7]
8	47/M	Primary	Dysuria and subsequent hematuria	Schindler et al., 1999 [16]
9	66/M	RT 4 years prior to Dx	Hematuria	Seethala et al., 2006 [8]
10	83/F	RT 14 years prior to Dx	Microhematuria	Kulaga et al., 2007 [17]
11	71/M	RT 10 years prior to Dx	Hematuria	Williams et al., 2008 [18]
12	32/F	Primary	Flank pain, hematuria	Warne et al., 2011 [2]
Current	89/M	RT 13 years prior to Dx	Hematuria	Bahouth, 2015

**Table 2:** Histopathology and immunohistochemistry of all patients reported.

Patient	Stage	Histology	Immunophenotype	Author/year
1	N/A	Classic, focally with large dilated vascular spaces	N/A	Jungano, 1907 [10]
2	N/A	Classic, focally solid	N/A	Casal et al., 1970 [11]
3	At least lamina propria	Typical, “hobnail” cells	fVIII+, keratin−	Stroup and Chang 1987 [12]
4	T3	Typical	fVIII+, keratin−	Aragona et al., 1991 [13]
5	T2	Typical	N/A	Ravi, 1993 [14]
6	T3	Typical	fVIII+, CD34+	Navon et al., 1997 [15]
7	T4N0M0	Solid and primitive	fVIII+, CD31+, CD34+	Engel et al., 1998 [7]
8	TXN + M0	Solid, focally classic	fVIII−, CD34−, keratin−, CD31+	Schindler et al., 1999 [16]
9	Peritoneal surface	Solid, focally primitive	CD31+, CD43+, kertin−	Seethala et al., 2006 [8]
10	Muscularis	Typical	CD31+, CD34−, fVIII−	Kulaga et al., 2007 [17]
11	T4	Typical	fVIII+, CD31+, CD34+	Williams et al., 2008 [18]
12	MIBC	Typical	fVIII+, CD31+, CD34+	Warne et al., 2011 [2]
Current	MIBC	Typical	CD31+, CD34+, fVIII+, keratin−; cystitis cystica	Bahouth, 2015

Typical appearance includes anastomosing vascular channels. N/A: not available. MIBC = muscle-invasive bladder cancer.
